# Establishment of mouse models for severe pulmonary hypertension through ‘double‐hit’ strategies

**DOI:** 10.1113/EP091833

**Published:** 2024-09-27

**Authors:** Lingdan Chen, Xin Chen, Yuhang Huang, Zhuoji Ma, Xiaohui Zeng, Tao Wang

**Affiliations:** ^1^ State Key Laboratory of Respiratory Diseases, Guangdong Key Laboratory of Vascular Diseases, National Clinical Research Center for Respiratory Diseases, Guangzhou Institute of Respiratory Health the First Affiliated Hospital of Guangzhou Medical University Guangzhou Guangdong China; ^2^ Department of Respiratory Medicine, Zhujiang Hosptial Southern Medical University Guangzhou Guangdong China

**Keywords:** hypoxia, mouse model, pneumonectomy, pulmonary artery ligation, pulmonary hypertension

## Abstract

Mouse models are crucial for understanding pulmonary hypertension (PH) mechanisms and developing therapies, but existing mouse models under hypoxia only exhibit mild PH. To address this, we established a double‐hit model combining unilateral pneumonectomy (LPx) or left pulmonary artery ligation (LPAL) with hypoxia exposure in C57BL/6 mice. Our detailed haemodynamic and histological evaluations post‐surgery demonstrated pronounced elevations in right ventricular systolic pressure (RVSP) (LPAL: 41.1 ± 4.63 mmHg, *P* = 0.005; LPx: 38.4 ± 2.95 mmHg, *P* = 0.002; Sham: 32.1 ± 2.21 mmHg) and pulmonary vascular wall thickness (LPAL: 56.9 ± 3.34%, *P* = 0.02; LPx: 54.3 ± 4.65%, *P* = 0.04; Sham: 44.8 ± 3.76%) compared to hypoxia‐exposed sham‐operated controls, reflecting a more severe PH phenotype. These novel models, which exhibit haemodynamic alterations akin to the established hypoxia with SU5416‐induced PH model as per published data, could offer a substantial contribution to future PH research and therapeutic development.

## Abstract (highlights)

- **What is the central question of this study?**
  Existing mouse models of pulmonary hypertension (PH) under hypoxia exhibit only mild PH: can a mouse model of PH be established that exhibits a more severe PH phenotype?
- **What is the main finding and its importance?**
  Mouse models with more severe PH were successfully established through a combination treatment involving unilateral pneumonectomy or left pulmonary artery ligation followed by hypoxia exposure. This approach results in a more pronounced PH phenotype compared to traditional models that rely solely on hypoxia exposure.

## 1 INTRODUCTION

Pulmonary hypertension (PH) is a critical condition defined by elevated pulmonary vascular resistance and pulmonary artery pressure, potentially culminating in right heart failure and death (Hassoun, 2021; Taichman et al., 2023). Animal models are instrumental in deciphering the pathophysiology of PH and underpinning the development of innovative treatments (Humbert et al., 2021). Although severe pulmonary vascular remodelling PH models have been successfully established in rats and larger mammals (Dignam et al., 2022), the most frequently utilized laboratory animal, the mouse, typically exhibits only mild PH when subjected to hypoxic conditions – a common method for creating mouse models of PH (Boucherat et al., 2022; Dignam et al., 2022; Gomez‐Arroyo et al., 2012; Sztuka & Jasińska‐Stroschein, 2017). To enhance the fidelity of PH in mice, our study introduces a novel approach by developing double hit models that integrate either unilateral pneumonectomy or left pulmonary artery ligation (LPAL), followed by hypoxic exposure, thereby intensifying the disease phenotype. This methodological advancement aims to provide a more robust platform for investigating PH mechanisms and for the discovery and testing of new therapeutic interventions.

## 2 METHODS

### 2.1 Ethical approval

The study was conducted in strict compliance with the ethical guidelines and approval from the Animal Care and Use Committee of Guangzhou Medical University (Approval No. 2022–71). We are committed to minimizing animal distress and ensuring their welfare throughout the experimental procedures.

### 2.2 Animals and surgical procedure

**FIGURE 1:**
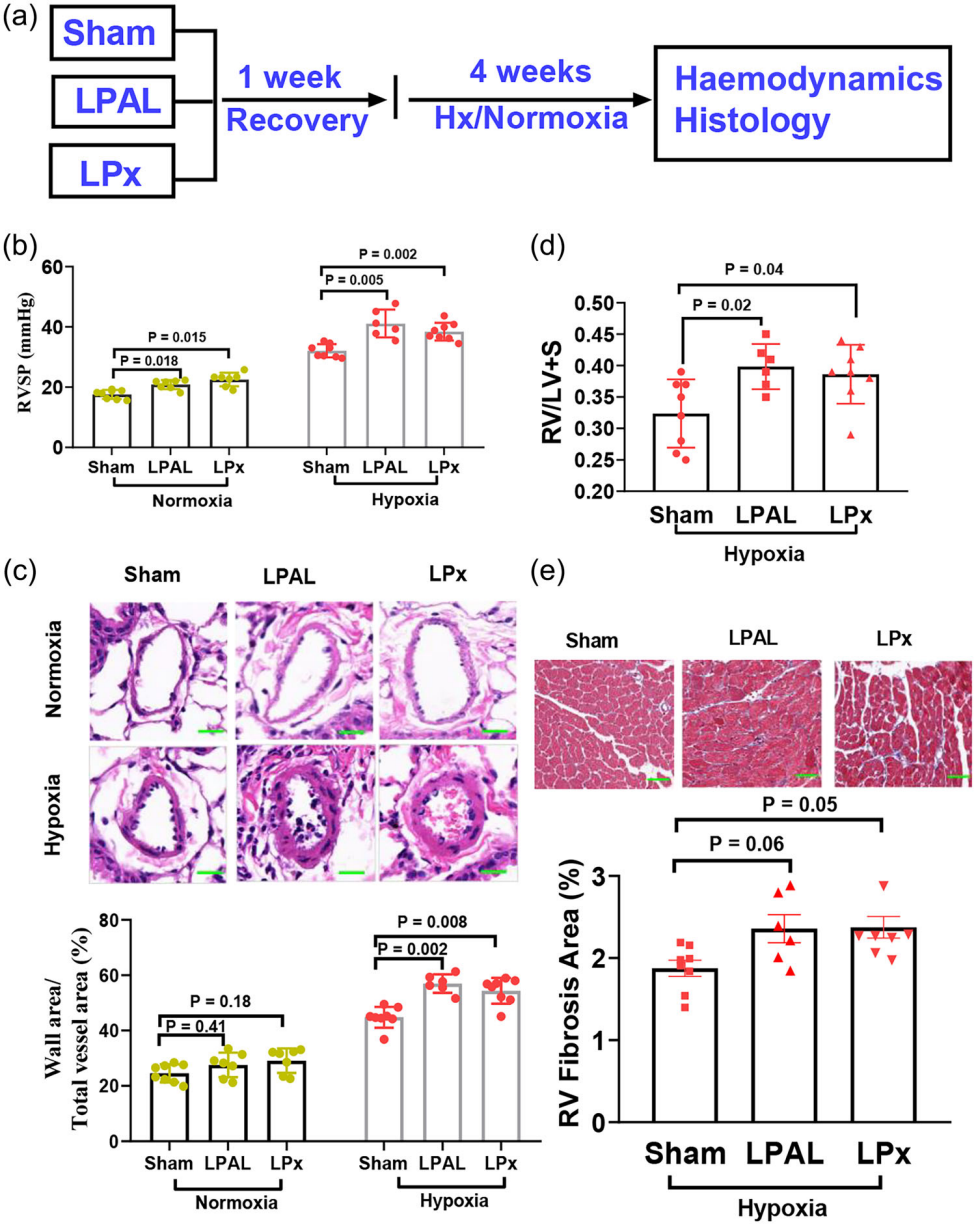
Enhancement of pulmonary hypertension by left pulmonary artery ligation (LPAL) and left pneumonectomy (LPx) under hypoxic conditions in C57BL/6 mice. (a) Experimental design: an illustrative overview of the study's procedural layout. (b) Both LPAL and LPx under either normoxic (21% O_2_) or hypoxic (10% O_2_) conditions lead to an increase in RVSP in C57BL/6 mice. (c) Both LPAL and LPx increase pulmonary vascular wall thickness in C57BL/6 mice under hypoxia, but do not change pulmonary vascular wall thickness in C57BL/6 mice under normoxia. (d) The Fulton index, a measure of right ventricular hypertrophy (RV/LV + S), is elevated in C57BL/6 mice subjected to LPAL or LPx under hypoxic conditions. (e) Neither LPAL nor LPx modifies right ventricular fibrosis (RV fibrosis was quantified by the ratio of the fibrotic area (stained blue) to the total area of the RV free wall, as revealed by Masson's Trichrome staining) when mice are exposed to hypoxia. Scale bar: 20 µm. Sham, sham‐operated; LPAL, left pulmonary artery ligation; LPx, left pneumonectomy; Hypoxia, 10% O_2_ exposure for 4 weeks; Normoxia, 21% O_2_ exposure; LV, left ventricle; S, septal; RV, right ventricle; RVSP, right ventricular systolic pressure.

Male C57BL/6 mice aged 6∼7 weeks were purchased from Guangdong Provincial Medical Experimental Animal Center (Guangzhou, China). Mice were randomly divided into six groups (*n* = 6∼8/group): sham‐operated followed by hypoxia (10% O_2_), left pneumonectomy (LPx) followed by hypoxia, LPAL followed by hypoxia; sham‐operated followed by normoxia (21% O_2_), LPx followed by normoxia, and LPAL followed by normoxia. After being anaesthetized by isoflurane (1∼3%) inhalation, mice were intubated and attached to a ventilator (HX‐101E, Techman, Chengdu, China). LPAL or LPx was performed under left thoracotomy. Sham operations mimicked the surgical process without actual ligation or resection. Anaesthesia depth was monitored by the response to the tail pinch; the absence of a response confirmed adequate anaesthesia. Throughout the surgery, heart rate and respiratory rate were closely monitored. Following a 1‐week recovery period under normoxic conditions, mice were exposed to either hypoxic or normoxic environments for 4 weeks (as depicted in Figure 1a). Post‐operative analgesia was administered via acetaminophen‐supplemented water (5 mg/mL, cat. no. 13200001166, Shanghai Johnson‐Johnson Pharmaceuticals, Ltd, Shanghai, China) for seven consecutive days. All animals were maintained in a specific pathogen‐free environment with ad libitum access to water and food.

### 2.3 Haemodynamics and histology

Haemodynamic measurements and histological evaluations were conducted 5 weeks post‐surgery, as detailed in a previous publication (Huang et al., 2022). Under anaesthesia, right ventricular systolic pressure (RVSP) was measured using a 1.2‐F micro‐tip pressure transducer catheter (Millar Instruments, Houston, TX) connected to a PowerLab data acquisition system (AD Instruments, Shanghai, China). Lung and heart tissues were immersed in 10% formaldehyde for subsequent staining with haematoxylin and eosin (HE) or Masson's Trichrome. A blinded observer evaluated pulmonary vascular wall thickness in 16–20 randomly selected intra‐acinar arteries per lung, with diameters ranging from 50 to 100 µm. The degree of wall thickening was expressed as the ratio (%) of the vascular wall area to the total vascular area. Right ventricular (RV) hypertrophy was determined using the Fulton index, which is the weight of the right ventricle normalized to the combined weight of the left ventricle and septum (RV/[LV + S]). RV fibrosis was quantified by the ratio of fibrotic area to the total area of the RV free wall, as revealed by Masson's Trichrome staining. At the conclusion of the experiments, mice were humanely euthanized using carbon dioxide (CO_2_) asphyxiation, a method recognized for its rapid induction of unconsciousness and subsequent euthanasia.

### 2.4 Statistical analysis

Data are presented as means ± standard deviation (SD). Group comparisons were performed using a two‐way analysis of variance (ANOVA) followed by Dunnett's post‐hoc test when comparing the effects of two independent variables, or a one‐way ANOVA followed by the Bonferroni post‐hoc test where only one independent variable was considered. A *P*‐value of less than 0.05 was considered to indicate statistical significance.

## 3 RESULTS

All mice survived the experimental procedures. Among mice exposed to normoxia, both LPAL (20.8 ± 1.53 mmHg, *n* = 6, *P* = 0.018) and LPx (22.5 ± 2.24 mmHg, *n* = 8, *P* = 0.015) led to a modest increase in RVSP compared to sham‐operated controls (Sham: 17.5 ± 1.47 mmHg; *n* = 8, Figure 1b), without a significant alteration in the pulmonary vascular wall thickness (Figure 1c). As expected, 4 weeks of hypoxic exposure elevated RVSP and pulmonary vascular wall thickness in all groups when compared to mice under normoxia exposure (Figure 1b,c). Notably, under hypoxic conditions, LPAL and LPx induced a pronounced increase in RVSP (LPAL: 41.1 ± 4.63 mmHg, *n* = 6, *P* = 0.005; LPx: 38.4 ± 2.95 mmHg, *n* = 8, *P* = 0.002; Sham: 32.1 ± 2.21 mmHg, *n* = 8; Figure 1b) and pulmonary vascular wall thickness (LPAL: 56.9 ± 3.34%, *P* = 0.02; LPx: 54.3 ± 4.65%, *P* = 0.04; Sham: 44.8 ± 3.76%; Figure 1c). The right ventricle's adaptation to pressure overload is a critical determinant of PH prognosis. Our findings indicate that both LPAL and LPx significantly enhanced RV hypertrophy (Figure 1d), yet did not induce significant RV fibrosis in hypoxia‐exposed mice (Figure 1e). These results suggest that the combined surgical and hypoxic approach effectively models severe PH manifestations in mice.

## 4 DISCUSSION

Here, we have successfully developed mouse models of PH that present more severe manifestations than those typically observed in traditional hypoxia‐induced PH models. Our innovative approach combines hypoxia exposure with a reduction in the pulmonary vascular bed, achieved through surgical removal of the left lung or ligation of the left pulmonary artery. This is significant because unilateral pneumonectomy is a procedure performed in cases of lung cancer, and pulmonary artery obstruction is a complication seen in patients with pulmonary embolism or vasculitis (Potaris et al., 2014; Yang et al., 2023). Our findings suggest that patients with these conditions may be more susceptible to the effects of hypoxia and should consider avoiding residence in high‐altitude, hypoxic environments.

To generate animal models of PH, increased blood flow velocity, which can lead to elevated vascular pressure, has been induced through pulmonary artery obstruction and pneumonectomy. In large animals, PH accompanied by RV hypertrophy has been induced in sheep through the combination of LPAL and progressive main pulmonary artery banding (Ukita et al., 2021). Similarly, PH was induced in piglets by LPAL followed by transcatheter embolization of the right lower‐lobe arteries (Mercier et al., 2013). These findings suggest that unilateral obstruction of the left pulmonary artery alone may not be sufficient to induce PH, which is consistent with our observations that LPAL or LPx under normoxia results in only a mild increase in pulmonary artery pressure without significant pulmonary vascular remodelling in mice. Consequently, more invasive strategies have been explored to elicit a more pronounced PH phenotype. These include right pulmonary artery ligation (Zhang et al., 2017) and extended pneumonectomy – where the left lung and right caval lobe are removed simultaneously (Tsikis et al., 2023). Compared to LPAL or LPx, these methods are more invasive and aim to disrupt a larger vascular bed. While the PH induced by right pulmonary artery ligation is similar to that observed in traditional hypoxia‐induced PH models, it is relatively mild and may not fully simulate the severity of human PH. Therefore, a ‘double hit’ approach that combines LPAL or LPx with hypoxia is proposed as a less invasive yet more effective method to generate a more severe PH phenotype.

Mice are favoured as model organisms due to their low cost and ease of breeding. However, the hypoxia‐induced PH models commonly used in mice only exhibit mild pulmonary vascular remodelling and a slight increase in RVSP. To overcome this limitation, various strategies, including genetic modification and pharmacological interventions, have been employed to intensify the pathological and haemodynamic features of PH in mice. For example, treatment with SU5416, an inhibitor of vascular endothelial growth factor, over a 3‐ to 4‐week period of hypoxia, has been shown to induce a higher RVSP compared to hypoxia alone (Vitali et al., 2014). Genetically modified mice, such as those overexpressing interleukin‐6 (IL‐6) or S100A4/Mts1 (Greenway et al., 2004; Steiner et al., 2009), or those with loss‐of‐function mutations in bone morphogenetic protein receptor type II (BMPR2) or prolyl hydroxylase domain enzyme 2 (PHD2) (Dai et al., 2016; Sztuka & Jasińska‐Stroschein, 2017), also develop a more severe form of PH under hypoxic conditions. Among these modified mouse models of PH, the combined treatment of SU5416 and hypoxia results in a relatively high RVSP (Boucherat et al., 2022), with a mean level of approximately 45 mmHg, similar to the ‘double‐hit’ models combining LPAL or LPx with hypoxia models in our study. A recent study reported a mouse model with severe PH using double knockout of both *Sirt3* (sirtuin 3) and *Ucp2* (uncoupling protein 2) genes; these mice developed plexiform‐like lesions in the pulmonary vasculature, which are pathological changes observed in severe PH patients, and exhibited a mean pulmonary artery pressure of about 50 mmHg (Zhang et al., 2021), which is higher than the models in our study. Unlike these mouse PH models, our approach uses surgery to reduce the vascular bed or lung surface area, rather than blocking or enhancing a single molecular signalling pathway. This method may avoid the interference of certain molecular signalling pathways in PH animal studies, as these pathways are critical to the development and progression of PH. PH is a multifactorial disease, and the models we have developed could represent a combination of group 3 (hypoxia) and group 4 (pulmonary artery obstruction). In clinical practice, patients with these conditions benefit from targeted PH drugs (Mirrakhimov & Strohl, 2016; Yang et al., 2023). These may implicate that our mice PH models are suitable for the development of novel PH therapies.

### 4.1 Conclusion

In summary, we have successfully created novel mouse models of PH by combining LPAL or LPx with hypoxia exposure, resulting in a more severe PH phenotype than that induced by hypoxia alone. These models offer a promising avenue for future animal studies and may pave the way for the discovery of new treatments for PH.

## AUTHOR CONTRIBUTIONS

All authors made substantial contributions to the conception and design of the work. Lingdan Chen, Xin Chen, Yuhang Huang, Zhuoji Ma, and Xiaohui Zeng: Performed Animal experiments; analysis and interpretation of data; and drafted the article. Tao Wang: Conception, Interpretation of data; revised the article. All authors have read and approved the final version of this manuscript and agree to be accountable for all aspects of the work in ensuring that questions related to the accuracy or integrity of any part of the work are appropriately investigated and resolved. All persons designated as authors qualify for authorship, and all those who qualify for authorship are listed.

## CONFLICT OF INTEREST

None declared.

## Data Availability

All data supporting the results in the paper are in the paper itself.
